# Virtual versus jaw simulation in Oral implant education: a randomized controlled trial

**DOI:** 10.1186/s12909-020-02152-y

**Published:** 2020-08-18

**Authors:** Baoping Zhang, Sihong Li, Shuting Gao, Mingfang Hou, Hong Chen, Lulu He, Yiting Li, Yumeng Guo, Errui Wang, Rui Cao, Jingyang Cheng, Ruiping Li, Kailiang Zhang

**Affiliations:** 1 School of Stomatology Lanzhou University, Lanzhou, 730000 China; 2 College of Medicine, Lanzhou University, Lanzhou, 730000 China; 3 Hospital of Stomatology, Lanzhou University, Lanzhou, 730000 China

**Keywords:** Dental education, Theoretical knowledge grade, Implant accuracy examination, Virtual reality model, Preclinical implant training

## Abstract

**Background:**

This research aims to investigate the evaluation methods of teaching oral implant clinical courses and estimate the effectiveness of a virtual simulation platform.

**Methods:**

Eighty second- and third-year undergraduates in Lanzhou University were recruited and randomized to either three experimental groups or one control group. The subjects undertook theoretical examinations to test their basic level of knowledge after training in similarly unified knowledge courses. Each student group then participated in an eight-hour operating training session. An operation test on pig mandible was conducted, followed by a second theoretical examination. The assessment consists of three distinct parts: a subjective operating score by a clinical senior teacher, an implant accuracy analysis in cone-beam computed tomography (angular, apical, and entrance deviation), and comparison of the two theoretical examinations. Finally, students completed a questionnaire gauging their understanding of the virtual simulation.

**Results:**

There was no significant difference between the four groups in first theoretical examination (*P* > 0.05); the second theoretical scores of the V-J and J-V group (62.90 ± 3.70, 60.05 ± 2.73) were significantly higher than the first time (57.05 ± 3.92, *P* < 0.05), while no difference between the V (57.10 ± 3.66) and J (56.89 ± 2.67) groups was found. Thus, the combination of V-J was effective in improving students’ theoretical scores. The V-J and J-V groups had higher scores on operation (73.98 ± 4.58, 71.85 ± 4.67) and showed better implant precision.

**Conclusion:**

Virtual simulation education, especially with a jaw simulation model, could improve students’ implantology achievements and training. Currently study found that the V-J group may performed better than the J-V group in oral implant teaching.

## Background

Digital technologies are rapidly advancing across different and diverse fields. One such promising and widely discussed digital technology is augmented and virtual reality. Augmented reality creates a virtual images and overlays them on a real environment, whereas virtual reality is a digital recreation of reality [1]—both these techniques require the “creation of reality” through computer graphics. In the medical industry, these computerized navigation devices enable its users to aggregate and visualize various medical data. For example, users can employ the helmet display to view medical data and images for surgery [2]. Similarly, doctors are applying virtual reality to treat mental health disorders [3, 4], eating disorders [5], and congenital heart disease [6] as well as for drug discovery [7]. Virtual reality has become especially popular in the dental sciences, such as in clinical trials for maxillofacial surgery protocols, human anatomy studies [8], treatment of patients with dental phobia [9], dental anesthesia training [10], and evaluation of endodontic surgery [11].

It is important to acknowledge that digital treatment and training, particularly virtual reality, are set to become mainstays in dentistry as well in the broader clinical and medical industry [1]. This trend is expected to greatly challenge conventional dental clinical practice and learning methods. As oral implant restoration technology advances, one crucial question deals with how to improve the teaching quality of oral implant education in order to train more professional implant clinicians. Previous studies in Europe and Australia showed that, with the integration of implant dentistry in daily dental practice [12, 13], more universities were including oral implant-related education in their undergraduate courses. Another study in India shows that 91.7% of students are eager to learn more about oral implants in college courses [14]. Thus, implant dentistry is slowly become a necessary part of pre-clinical teaching in dental education.

Currently, there is immense regional or geographical heterogeneity in implant education [15]; in different countries, the characteristics of the “basic,” “intermediate,” “advanced,” and “specialist” stages differ [16]. In China’s dental implant education system, the simulation laboratory model is considered common, but important, tools for oral experimental teaching and pre-clinical skills training, these tools require specialized equipment and are costly [17]. However, the current model is simple; it cannot simulate difficult cases in actual clinical practice. Thus, only the more experienced trainers are likely to perform complex clinical procedures [18]. In this regard, digital treatment and training that employs virtual reality offer a novel direction in training for dental implant surgery.

The major benefit of applying reality technologies to dental education is in pre-clinical skills training. Students can acquire the necessary skills through repeated digital practice. Virtual reality simulation also gives standardized feedback, which is critical for targeted guidance to students [19]. However, only few applications of virtual simulation technology exist in oral implant surgery training. Thus, the full effect of virtual simulation on such training is inconclusive. Only further research can reveal the best approach and time to incorporate virtual simulation in training programs.

By evaluating the theory and operation procedures of dental students/doctors of implantology, the current study compares the effects of pre-clinical training using a jawbone model, a virtual simulation system, and different orders of a virtual simulation system combined with the jawbone model. This way, current study could find the best approaches to introducing reality technology in implantology training, improve teaching programs, and gauge the effect of such an education in clinical practice.

## Methods

### Participants

For this study, we selected 80 s- and third-year undergraduate students of stomatology at Lanzhou University. The random setting as follow: total of 166 students were divided into two groups (male/female) firstly according to gender. Then 40 students were randomly selected in each of two packets using a random-number table consisted of 80 participants ultimately, which can draw random sampling scientifically. The subjects were randomly divided into four groups (*n* = 20) with a similar male-to-female ratio following CONSORT guidelines. The four groups included one control group and three experimental groups: (1) jaw simulation model as control, (2) virtual simulation, (3) virtual-jaw (V-J), (4) jaw-virtual (J-V). The age distribution of the participants was in the range of 18 to 21 years with no regional differences. None of the subjects had received oral implantology courses before this study. Informed consent was obtained from all participants and the study protocol was approved by the Ethics Committee, School of Stomatology, Lanzhou University.

### Study procedure

#### Theory teaching of implantology

**Fig. 1:**
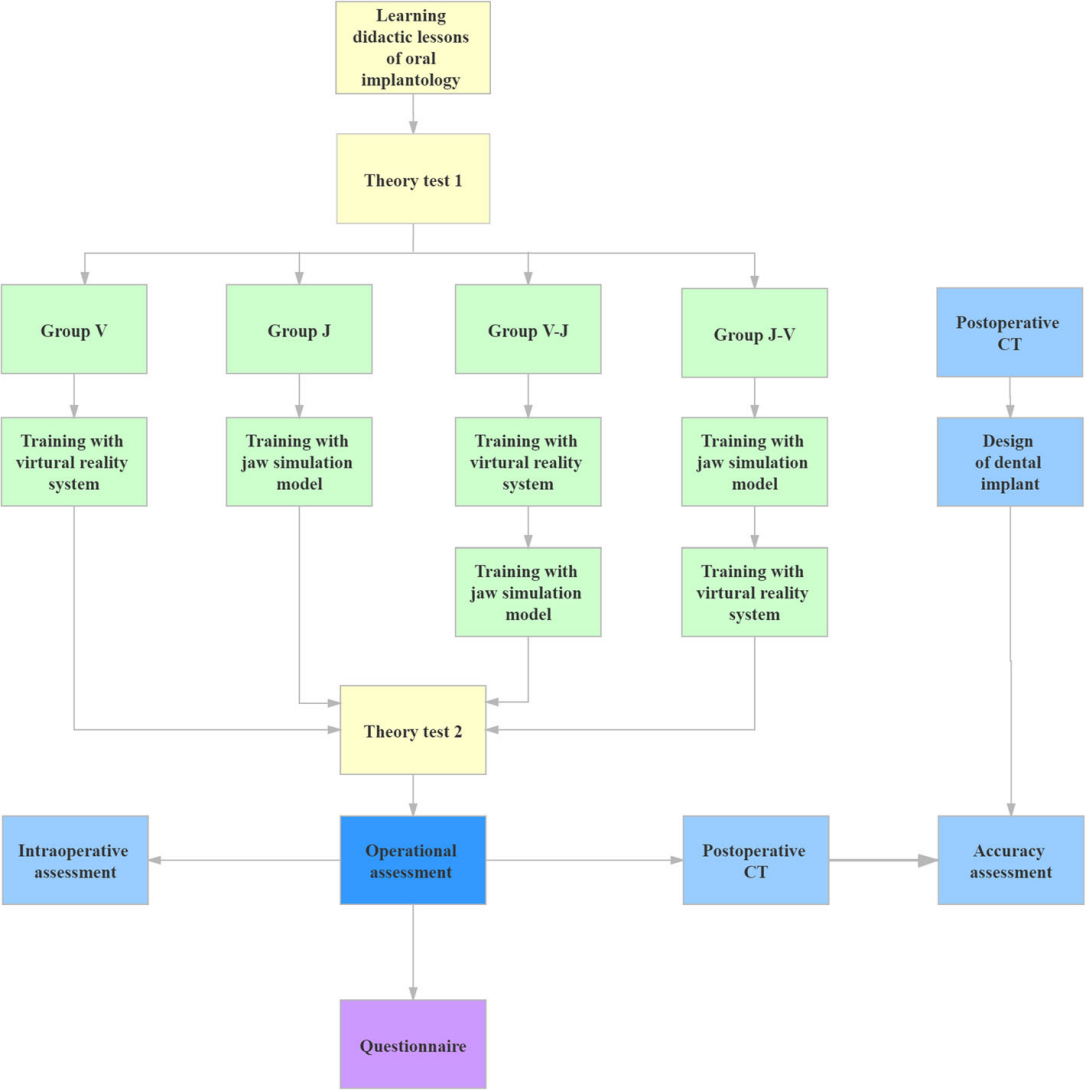
Flow diagram of the process using the virtual reality system and the jaw simulation model for implant

Before receiving the operation training, all students participated in the theory courses and underwent initial assessment (See Fig. 1 for the flow chart). Each lecture was 2 h long and taught by instructors with more than 10 years of clinical experience. The theoretical content covered implant apparatus, preoperative preparation, basic principles of implant, applied anatomy, and operative procedure, and based on *Oral Implantology*, second edition [20]. The first theory exam was a multiple-choice questionnaire taken by all participants.

#### Implanting operation teaching

**Fig. 2:**
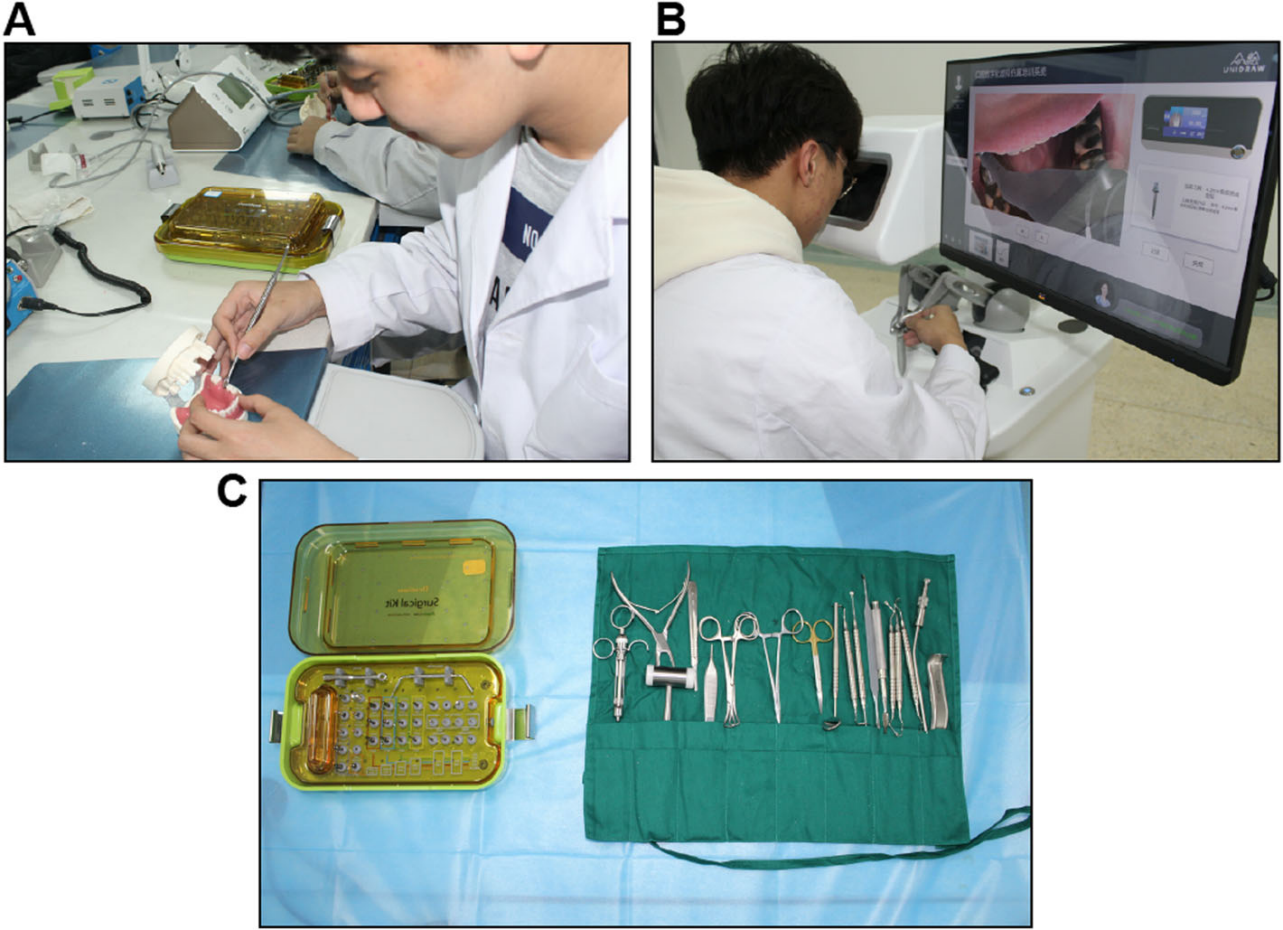
Training process of implant operation (**a**) Training with jaw simulation model (**b**) Training with virtual reality system (**c**) Implant tool box

The operation training lasted 8 h over 4 days. The site of operation was the mandibular molars recreated on a virtual simulation system (Zhonghui Technology Institute, UniDental-MS01, China) and jaw simulation model (Fig. 2a, b). The implant systems followed the Dentin implant surgical operation manual [21] and each group used a dental implant toolbox (Fig. 2c). The order of the V-J and J-V groups was the opposite, that is, the eight-hour long operation was split into two four-hour operations for each group. This way, we evaluated the operational training sequence on effect of teaching.

#### Preparation before examination

**Fig. 3:**
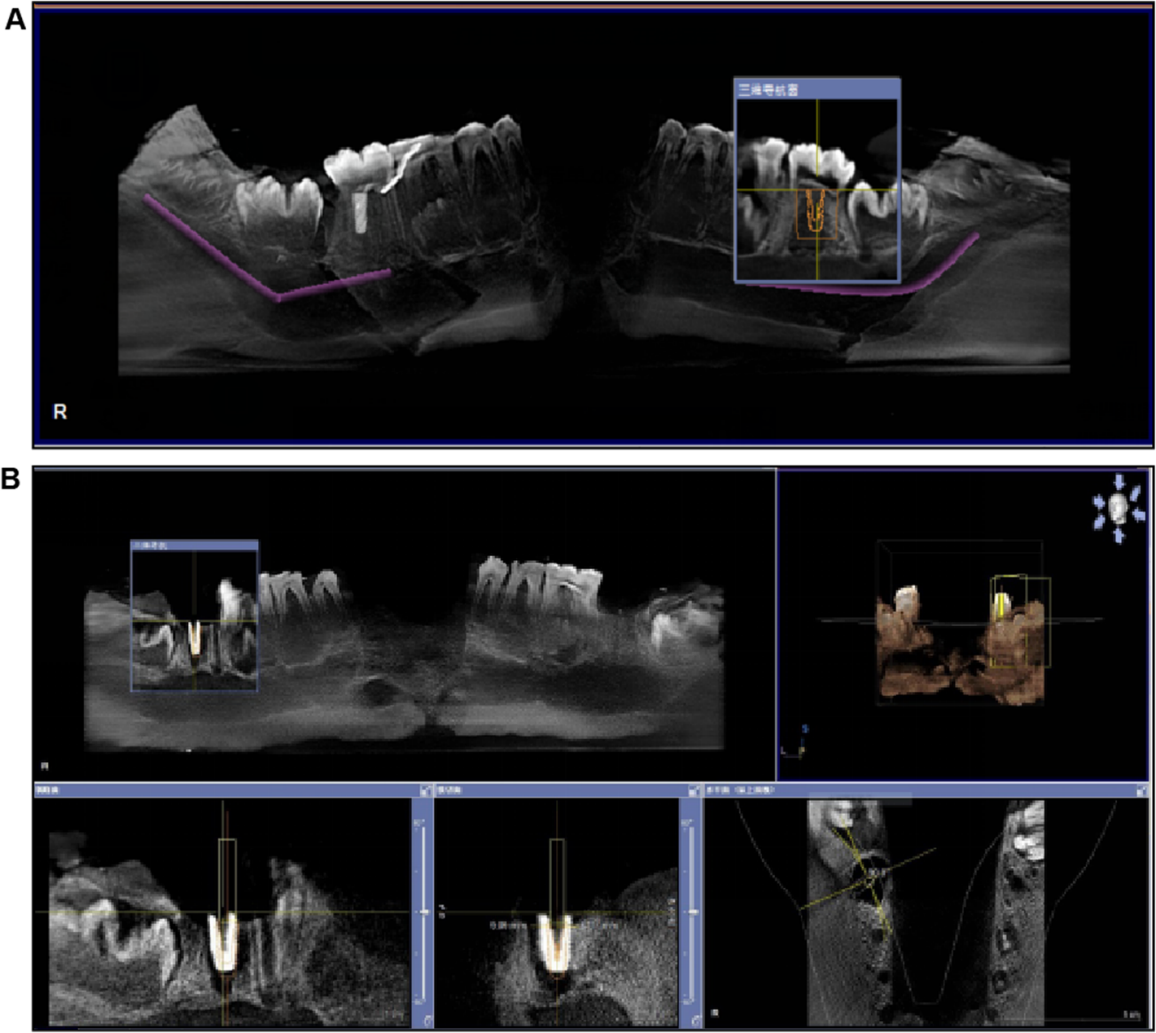
Analysis with GALILEOS Viewer software (**a**) Planned implant design (**b**) Deviation of the placed implant

The mandible of a pig (Yorkshire) was scanned using cone-beam computed tomography (CBCT). The imagine data were imported into a software (Sirona Dental, GALILEOS Viewer, German). According to the bone condition and anatomical structure, the best implantation site on the mandibular second molar was designed by the teachers (Fig. 3a). Subsequently, the ideal site was assessed for deviation from the actual site (Fig. 3b).

#### Operational assessment and secondary examination

In the participants’ assessment, the second mandibular molars of a pig were extracted as the implant site. The assessment was double-blinded and evaluated by professional dentists, it included two parts: (1) a subjective evaluation with students’ “preoperative preparation” and “intraoperative operation” procedures (See Table 1 for details on the evaluation); (2) an objective evaluation of the images obtained using the CBCT. The former part focused on the standardization and professionalism of the operations, while the latter part was used to judge the effects of implanting. Then, a second theoretical test was conducted to analyze the differences between the first and second testing scores and, thus, determine whether operation training could improve students’ theoretical knowledge.

**Table 1:** Planting operation score sheet

Scoring items	Score
***Preoperative preparation***	
Dress neatly, asepsis, necessary preoperative instructions	5
Assessing implant conditions and designing surgical procedures	5
Choosing surgical instruments according to the surgical plan	5
***Intraoperative operation***	
**Incision**	
● Suitable length	3
● Avoiding damage to adjacent tissues	3
● Minimally invasive principle	3
**Dressing the alveolar ridge**	
● Ensuring the height of the alveolar ridge	3
● Ensuring the width of the alveolar ridge	3
**Handling planting nest**	
● Locating the plantation nest	5
● Determining planting nest orientation	5
● Enlarging the diameter of the planting nest	5
● Formation of planting nests	5
● Flushing	5
**Implanting the implant**	
● Placing the implant	5
● Removing the carrier	5
● Closing the implant	5
● Closing the wound	5
***Overall effect***	
Implant position	5
Implant depth	5
Implant orientation	5
Distance between implant and adjacent teeth	5
***Total***	100

#### Implant accuracy

**Fig. 4:**
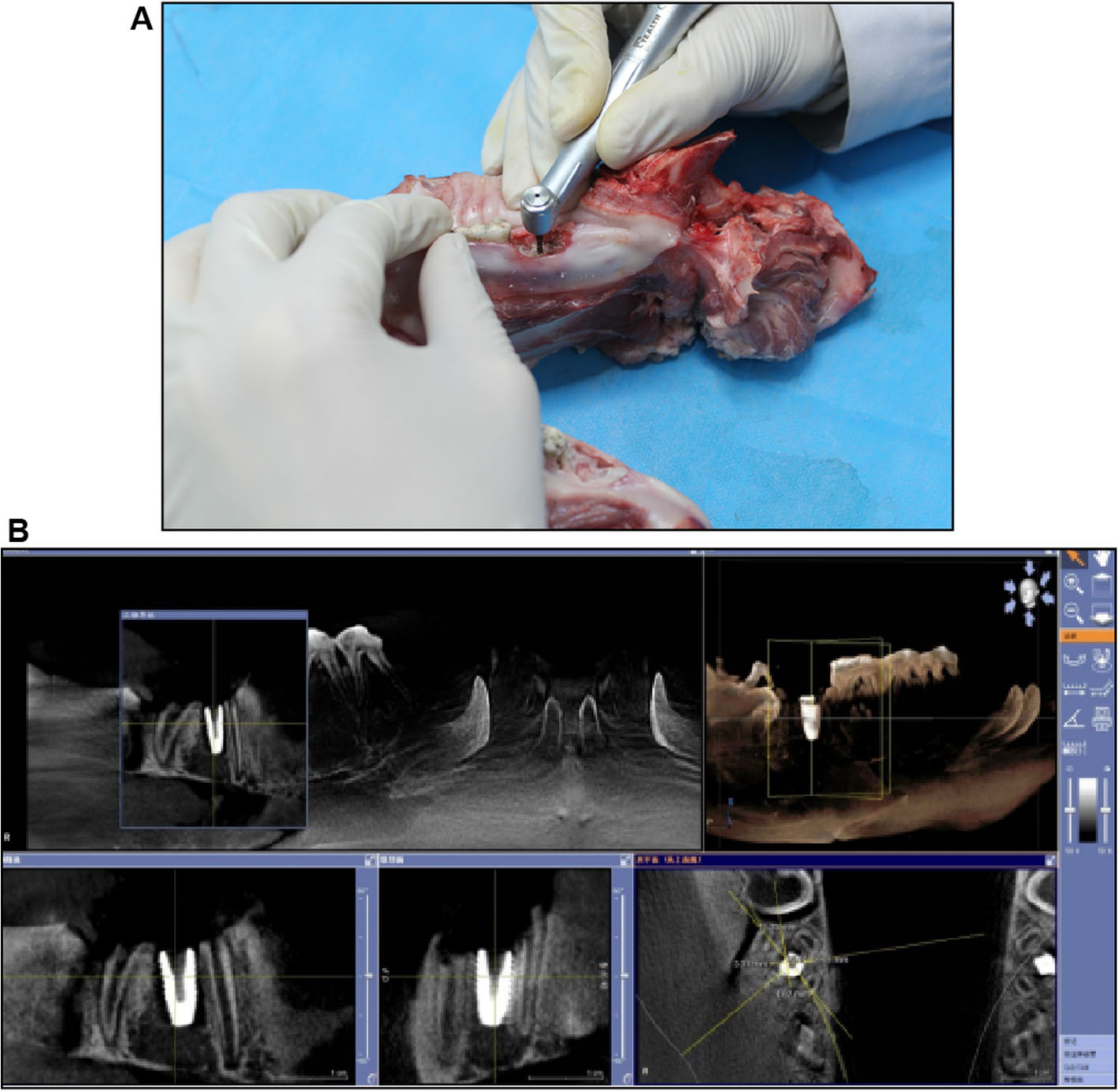
Implant operated evaluation (**a**) Subjective evaluation of the process (**b**) Objective analysis of implant accuracy

The CBCT scan of the implanted site and the imported data were used to evaluate the rationality of implanting in order to calculate the angular, apical, and shoulder deviations. (Fig. 4).

#### Questionnaire survey

The degree of satisfaction from the students was gathered through the questionnaire using a Likert scale after the teaching session [22]. Each item was rated, with 5 indicating “strongly agree,” 4 indicating “agree,” 3 indicating “neither agree nor disagree,” 2 indicating “disagree,” and 1 indicating “strongly disagree.”

#### Statistics analysis

A comparison between the four groups was done through an analysis of variance (ANOVA) in case the data conformed to normal distribution with homogeneous variance. For data not conforming to the normal distribution, a non-parameter test was adopted. The study compared the theoretical scores before and after the operation through a paired sample *t*-test. The operational assessment was based on a one-way ANOVA.

## Results

### Theoretical examination scores

**Fig. 5:**
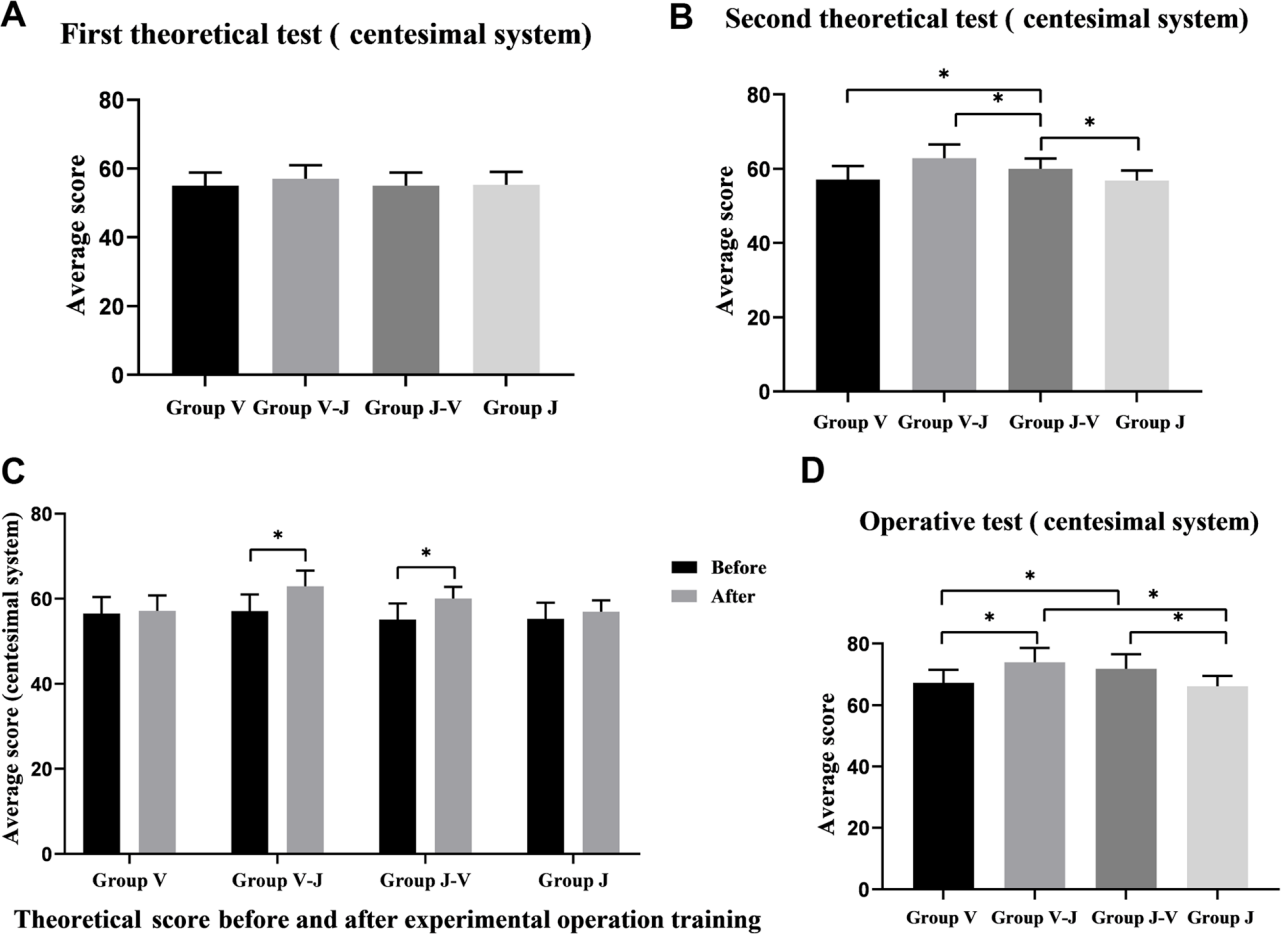
Test scores (**a**) Comparison of results on the first theoretical test (**b**) Comparison of results on the second theoretical test (**c**) Theoretical results of each group before and after operation training (**d**) The subjective evaluation result of operation test

The students’ first test average scores are shown in Fig. 5 A (*P* > 0.05); the results show no statistically significant difference for the V, J, V-J, and J-V groups (56.50 ± 3.88, 55.26 ± 3.79; 57.05 ± 3.92; 55.05 ± 3.80, respectively). Thus, the theoretical lectures imbibed students with similar power to implement their professional knowledge.

The average scores for the second exam after completing the full operation training are shown in Fig. 5b (V group: 57.10 ± 3.66; J group: 56.89 ± 2.67; V-J group: 62.90 ± 3.70; J-V group: 60.05 ± 2.73), the results show statistical significance in the difference (*P* < 0.001). The V-J group was found to be significantly superior to other groups, whereas the J-V group performed better than the J and V groups. Therefore, the investigation can be stated that operating on the virtual simulation system first would be more conducive to mastering knowledge. There was no difference between the V and J groups (*P* = 0.962).

The average difference scores for theory learning before and after the training indicate that the first score of the V-J group (57.05 ± 3.92) is lower than the second (62.90 ± 3.70, *P* < 0.001); we find similar results for the J-V group (55.05 ± 3.80; 60.05 ± 2.73; P < 0.001). However, the difference in the V and J groups was not significant (Fig. 5c). These results show that the model-based simulation fits better than the single one under the same training duration.

### Operational assessment

The assessment scores for the individual groups are reported in Table 2; the mean scores for each group are represented in Fig. 5d. The V-J and J-V groups (73.98 ± 4.58; 71.85 ± 4.67) show higher scores than the V and M groups (67.275 ± 4.24; 66.15 ± 3.40), with a statistically significant difference. No difference was found between the V-J and the J-V groups (*P* = 0.128). Similar trends were observed between groups V and J (*P* = 0.417).

**Table 2:** Operational assessment results

Scoring items	Score (Mean ± SD)			
	V	J	J-V	J-M
Preoperative preparation	10.65 ± 1.91	9.85 ± 0.94	11.60 ± 2.01	12.45 ± 1.65
Intraoperative operation	43.63 ± 3.50	43.05 ± 3.24	45.25 ± 3.51	45.63 ± 3.05
Overall effect	13.00 ± 1.58	13.25 ± 1.41	15.00 ± 1.45	15.90 ± 1.51
Total	67.28 ± 4.24	66.15 ± 3.40	71.85 ± 4.67	73.98 ± 4.58

**Fig. 6:**
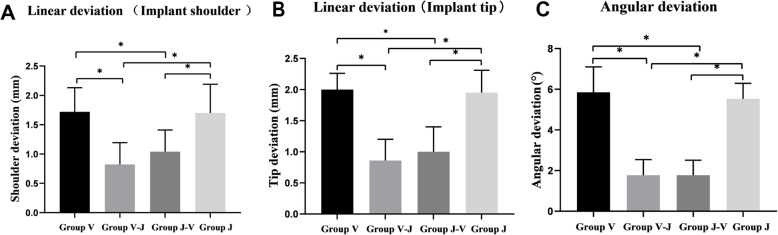
Comparison of the mean deviations of implants (**a**) Mean linear deviation of implant shoulder (**b**) Mean linear deviation of implant tip (**c**) Mean angular deviation of implant

Table 3 and Fig. 6 describethe experimental results of implant accuracy from the CBCT. The main overall effect in the V-J and J-V groups shows improvement and higher accuracy. That is, the combination of the two training methods helped students improve their implant clinical skills, but the order (whether V-J or J-V) is independent.

**Table 3:** Deviation of implant accuracy

Deviations	Groups (Mean ± SD)				*P* value
	V	J	V-J	J-V	
Linear deviation (mm)					
Implant tip	2.00 ± 0.26	1.95 ± 0.36	0.86 ± 0.34	1.00 ± 0.40	<0.001
Implant shoulder	1.72 ± 0.41	1.70 ± 0.49	0.82 ± 0.37	1.04 ± 0.37	<0.001
Angular deviation(°)	5.85 ± 1.25	5.53 ± 0.76	1.77 ± 0.77	1.77 ± 0.74	<0.001

### Questionnaire

Table 4 displays the student evaluations of the teaching methods; the evaluation score for the V and J groups is 3, that is, “general.” In the joint teaching unit, the score indicates 4 points, that is, “satisfied.” Data from the subjective feeling questionnaires show that the V-J and J-V groups had better teaching experience.

**Table 4:** Questionnaire survey

Project Evaluation Score	Groups (Mean ± SD)			
	V	J	J-V	J-M
Course focus	3.45 ± 0.50	3.15 ± 0.36	4.75 ± 0.43	4.70 ± 0.46
Course interest	3.80 ± 0.40	3.85 ± 0.36	4.80 ± 0.40	4.65 ± 0.48
Course richness	3.60 ± 0.49	3.40 ± 0.49	4.85 ± 0.36	4.80 ± 0.40
Acquisition of konwledge	3.30 ± 0.46	2.95 ± 0.22	3.90 ± 0.44	4.25 ± 0.43
Combine theory with practice	3.70 ± 0.46	3.35 ± 0.48	4.85 ± 0.36	4.60 ± 0.49
Improvement of clinical skills	3.80 ± 0.40	3.25 ± 0.43	4.70 ± 0.46	4.85 ± 0.36
The activity of the class atmosphere	3.35 ± 0.48	3.90 ± 0.30	4.85 ± 0.36	4.60 ± 0.49
Improvement of learning motivation	3.35 ± 0.48	3.20 ± 0.40	4.70 ± 0.46	4.85 ± 0.36
Satisfaction with the use of laboratory	3.60 ± 0.49	3.50 ± 0.50	4.85 ± 0.48	4.85 ± 0.36
Interaction between teachers and students	3.55 ± 0.50	3.05 ± 0.22	4.90 ± 0.30	4.55 ± 0.50

## Discussion

To achieve optimal methods of teaching oral implant clinical courses and productively applied virtual simulation platform. This study was seek to find a better teaching mode and avoid the disadvantages of traditional teaching. Thus, students can acquire the necessary and adequate skills before beginning their clinic practice.

The results of this study show that, compared with the effects of traditional training, a single virtual simulation system, or a single jaw simulation model, a combination of virtual reality simulation and jaw simulation model is better in terms of theory, operation as well as implant accuracy (shoulder deviation, root deviation, and angle deviation). Jasinevicius [23] had showed that students in contemporary non-computer-assisted simulation system groups took five times more time than students in virtual reality computer-assisted simulation system groups in the preparation of cavity and full gold crown. De Boer [24] also revealed that dental students could improve their operation dexterity skills by using the Simodont dental trainer to repeatedly practice different levels of force feedback training. Evidently, virtual and augmented reality technologies can promote enjoyment in learning and the acquisition of operational skills. Especially, virtual simulation systems exhibit great potential in dentistry teaching [25], which is consistent with our conclusion. However, the advantages of a pure virtual simulation system are not as obvious as in the prior research. This may be attributed to the different disciplines and equipment required in the virtual simulation system.

In our results, the V-J group made significant progress. Its students achieved higher theoretical scores by first using a virtual simulation system to familiarize themselves with the dental implant operation process and its main points. Then, they performed physical operations on the jaw model. Teachers believe such a combined education system is better because the virtual simulation technology can simulate the entire dental procedure as well as the corresponding environment—from the patient’s clinical admission to the end of the diagnosis and treatment [26]. Thus, students can quickly grasp the entire clinical implantation process. There is also immense theoretical knowledge instilled in the training process. Other virtual simulation systems can also give targeted feedback during training [27–29], such sustained feedback will maximize the final effect of virtual training [30].

In addition, we should consider students’ limited concentration [31], the theoretical knowledge of virtual reality systems should be taught first. Studies have also shown that gender differences can lead to differences in medical tests [32], and hence we grouped our participants to circumvent this difference. In the final evaluation of the implanting operation in this study, compared with other groups, the V-J and J-V groups showed higher average operation scores, the CBCT planting accuracy was also higher. These results show that our combined training method is conducive to mastering operation skills. However, the sequence of training does not affect the acquisition of implant operations. Although the virtual simulation system can perform screen construction and sensory stimulation in the operation process, the fidelity of its simulation of vision, hearing, and touch cannot be completely compared with actual reality. However, the jaw simulation model can compensate for shortcomings, making a combination system useful.

This research has three important practical significances for future oral implant teaching. First, instructors can use this study as a reference to modify new teaching methods by including virtual simulation systems and jaw simulation models into their teaching courses. This would improve the proficiency in implant skills of dental students. Seifert et al. similarly regarded virtual patient cases as an effective alternative to lecturer-led small group teaching [33]. Second, the use of the virtual simulation system can decrease faculty time in instruction and supervision. Our combined method can save time and human resources expended in implant teaching by reducing trainers’ workload. However, the guiding role of teachers cannot be completely replaced by technology. For instance, Lechermeier and Fassnacht [34] found that feedback from professionals of higher status and expertise was still the most effective in training. However, the cost of virtual simulation systems is lower when used for a long time [35]. Third, the result obtained in this survey show students reported greater levels of satisfaction for the combination teaching methods than the students assigned to the traditional teaching method condition. We speculate that they develop an appreciation of this teaching method and the value of acquiring clinical training skills.

Nevertheless, there are some limitations to this study, such as the training time was too short to fully predict the long-term effect of various teaching methods. Because the models of virtual simulation system equipment are not uniform across geographies, simulators use different training levels, they have different contexts and levels of difficulty [36]. Due to this limitation, the results of our experiment cannot explain the application value of all virtual simulation systems. Although the pig has proven to be a suitable bio-model for both research purposes and for training medical professionals because of its similarity to humans [37], the effect of using it for the evaluation of dental implant operations is still not completely equivalent to real clinical cases. Future studies should examine the long-term application effect of the virtual simulation system, especially its optimal application period in the teaching process, as these questions remain unclear, but significant. In addition, it is of great importance to develop more realistic virtual simulation equipment.

## Conclusion

The present preliminary study could provide an interaction training method for implantology, combines the virtual simulation system and the jaw simulation model has a positive effect on theoretical knowledge and pre-clinical skills.

## Data Availability

The datasets used and/or analyzed during the current study are available from the corresponding author on reasonable request.
